# Metformin treatment modulates the tumour-induced wasting effects in muscle protein metabolism minimising the cachexia in tumour-bearing rats

**DOI:** 10.1186/s12885-016-2424-9

**Published:** 2016-07-07

**Authors:** André G. Oliveira, Maria Cristina C. Gomes-Marcondes

**Affiliations:** Department of Structural and Functional Biology, Biology Institute, State University of Campinas, UNICAMP, Rua Monteiro Lobato, 255, CP 6109, Campinas, São Paulo 13083862 Brazil

**Keywords:** Cancer, Metformin, Protein synthesis, Protein degradation, Muscle metabolism, Walker-256 tumour

## Abstract

**Background:**

Cancer-cachexia state frequently induces both fat and protein wasting, leading to death. In this way, the knowledge of the mechanism of drugs and their side effects can be a new feature to treat and to have success, contributing to a better life quality for these patients. Metformin is an oral drug used in type 2 diabetes mellitus, showing inhibitory effect on proliferation in some neoplastic cells. For this reason, we evaluated its modulatory effect on Walker-256 tumour evolution and also on protein metabolism in gastrocnemius muscle and body composition.

**Methods:**

Wistar rats received or not tumour implant and metformin treatment and were distributed into four groups, as followed: control (C), Walker 256 tumour-bearing (W), metformin-treated (M) and tumour-bearing treated with metformin (WM). Animals were weighed three times a week, and after cachexia state has been detected, the rats were euthanised and muscle and tumour excised and analysed by biochemical and molecular assays.

**Results:**

Tumour growth promoted some deleterious effects on chemical body composition, increasing water and decreasing fat percentage, and reducing lean body mass. In muscle tissue, tumour led to a decreased protein synthesis and an increased proteolysis, showing the higher activity of the ubiquitin-proteasome pathway. On the other hand, the metformin treatment likely minimised the tumour-induced wasting state; in this way, this treatment ameliorated chemical body composition, reduced the higher activities of proteolytic enzymes and decreased the protein waste.

**Conclusion:**

Metformin treatment not only decreases the tumour growth but also improves the protein metabolism in gastrocnemius muscle in tumour-bearing rats.

## Background

Cancer is one of the leading causes of deaths worldwide [1], and many cancer patients show malnutrition and weight loss due to tumour-associated catabolic stress, developing cancer-cachexia state. Cachexia is a complex metabolic syndrome associated with others underlying illness, characterised by inducing lean body mass wasting, with or without fat mass loss [2]. Depending on the type of cancer, more than 50 % of cancer patients present cachexia, and this is one of the most frequent cause of morbidity and mortality [3]. In cancer cachexia, the major pathway responsible for muscle protein degradation is the ubiquitin-proteasome pathway (UPP) [4] which consists of a set of enzymes that couples target proteins to ubiquitin molecules, which are degraded by the 26S proteasome [5]. In tumour cells, the phosphoinositide 3-kinase (PI3K)–Akt–mammalian target of rapamycin (mTOR) pathway is always turned on due to mutation of key genes in this pathway. On the other hand, in muscle cells, this pathway is responsible mainly for control protein synthesis and degradation process [6]. Akt activation by insulin has been linked to suppression of FOXO (forkhead box O) and caspase-3 activity and also to a decrease in ubiquitin ligases expression [7]. In this way, many cancer patients experience insulin resistance in some extend, which in certain cases lead to an increased protein degradation by FOXO and higher UPP activity [7].

AMP-activated protein kinase (AMPK) plays an essential role maintaining the cellular energetic status, acting as a fuel gauge. This process helps cells to deal with energy stress conditions and bring them back to homoeostasis [8], controlling processes such as protein synthesis. Metformin (1,1-dimethylbiguanide) is an oral drug widely used in type 2 diabetes mellitus (T2DM) treatment, being prescribed to more than 120 million of people worldwide [8] and many studies shows that T2DM patients treated with metformin have a lower risk of developing cancer [9, 10]. The mechanism by which metformin act is not completely understood, but it seems to be through phosphorylation and activation of AMPK. However, this effect on AMPK activation is indirect and might be an inhibitory effect on mitochondrial respiratory chain complex 1 [11–13].

Despite the fact that metformin can decrease insulin levels and tumour’s cell proliferation [14], few studies analyse metformin action on skeletal muscle in cachexia models. Therefore, here we hypothesise that metformin could decrease the inhibitory effect on insulin signalling in muscle tissue caused by tumour growth.

## Methods

### Animals and tumour implant

Male Wistar rats weighted within 60–70 g (3 weeks old) were obtained from the animal facility CEMIB/State University of Campinas and housed in a collective cage, maintained at 22 ± 2 °C and 12 h light/dark cycles. Animals were allowed to a normoprotein semi-purified diet (18 % of protein source, following the AIN-93G recommendations) [15] and water ad libitum. Walker 256 cells were originally obtained from Christ Hospital (USA) and maintained in our laboratory through consecutive intraperitoneal passages; 1 × 10^6^ viable tumour cells, in 0.2 mL NaCl suspension, were implanted subcutaneously in the right flank, while the rats without tumour received 0.2 mL of 0.9 % NaCl injection as a sham procedure. The general UKCCCR [16, 17] guidelines were followed, and the experimental protocol (#895-1) was approved by the Ethical Committee of the State University of Campinas.

### Metformin treatment and experimental procedures

Metformin was dissolved in drinking water (100 mg/L) which corresponded to 33 mg/Kg body weight. Metformin concentration was estimated using the allometric scaling [18, 19] from human data, using the initial recommended dose for T2DM patients (500 mg/day). Animals were distributed into four groups: control (C), Walker 256 tumour-bearing (W), metformin-treated (M) and tumour-bearing treated with metformin (WM). Tumour weight was measured as previously described [20] and when tumour mass in W animals reached 10 % of body mass, the animals were subjected to overnight fast time (12 h) and euthanatized by cervical dislocation. A blood sample was collected by cardiac puncture. Carcass weight was obtained by weighing the animals without gastrointestinal tract and tumour mass. Both gastrocnemius muscle and tumour tissue were expressed as the percentage of the carcass weight.

**Biochemical body composition assay** was previously described [21]. The samples of dried and fat-extracted carcasses were analysed for total nitrogen spectrophotometrically [22]. Another sample was submitted to alkaline digestion for collagen nitrogen determination [22]. Non-collagen nitrogen was calculated by subtracting collagen nitrogen from total nitrogen.

### Serum proteins concentration

Total serum proteins and albumin were quantified spectrophotometrically [23] (Laborlab, Brazil), and the difference between total serum protein and albumin contents we calculated the serum globulin content.

### Gastrocnemius muscle sampling and enzymatic assays

Immediately after euthanasia, the gastrocnemius muscles were dissected, frozen in liquid nitrogen and kept at - 80 °C. A sample was then homogenised in phosphate buffer saline (PBS) (137 mM NaCl, 2.7 mM KCl, 10 mM Na_2_HPO_4_, 1.8 mM KH_2_PO_4_), pH 7.4, 0.1 % Triton X-100 and the supernatant was used for protein concentration [24] and all enzymatic activity assays. Calpain [25], cathepsin B and H [26] and chymotrypsin activity [27] were assayed as previously described without modification.

### Muscle protein degradation and synthesis

Right gastrocnemius muscle was dissected and incubated with 0.5 μCi/mL L-[2,3,4,5,6-^3^H] phenylalanine (Amersham, UK) for protein synthesis assay while left muscle was incubated with 0.5 mM cycloheximide for protein degradation assay. Radioactive phenylalanine incorporated into muscle and tyrosine released in incubation buffer were assayed as described [28]. Protein turnover was defined as the ratio of protein synthesis and protein degradation.

### Immunoblotting

Immunoblotting was performed as previously described [20]. Proteins were probed with primary antibodies GAPDH (sc-25778), Ubiquitin (sc-6085), UBE2A/B (sc-10479) (Santa Cruz Biotechnology, USA); 20S (PW8195), 19S (PW8165), 11S (PW8185) (Enzo-Biomol, USA); AMPK (2603), phospho-AMPKα^Thr172^ (2535), Akt (4685), phospho-Akt^Thr308^ (4056), goat anti-rabbit (7074), horse anti-mouse (7076) (Cell Signalling, USA) and rabbit anti-goat (AP106P) (MerckMillipore, USA). Images were captured using Alliance 2.7 (UVItec, UK) and band intensity was quantified using UVIband -1D (UVItec, UK).

### Statistical analysis

The nonparametric *t*-test was used for comparison between two groups. Comparison among groups, we used One-way ANOVA followed by Bonferroni post-test, using GraphPad Prism version 5.00 software (GraphPad, USA) and results showed as mean ± standard deviation (SD). Significance was defined as *p* < 0.05.

## Results

### Metformin treatment attenuates effects of tumour growth on body composition

To analyse whether tumour growth led to cachexia and whether metformin treatment, at close therapeutic dose used in T2DM patients, was able to reduce the tumour harmfull effect, we analysed the biochemical body composition parameters. As can be seen in Table 1, W animals developed cachexia. Although final body weight decreased in both tumour-bearing groups, the W group weight was approximately 10 % less than C rats, while the WM group had no difference compared to C. Body water content increased in both tumour-bearing groups; in the opposite way, the body fat reduced in W and WM groups. Lean body mass in W group was around 89 % of C group while the WM had similar value compared to C. Comparing both tumour-bearing groups, the results showed that WM had approximately 8 % lean body mass higher than W. In addition, carcass protein reduced in W (15 % lower) and WM (12 % less) in comparison to control group. The carcass collagen nitrogen also reduced by tumour effects, being 35 % lower in W group, while decreased around 13 % in WM group; this result showed that metformin treatment led to maintenance of collagen nitrogen in WM when compared to W group. The decrease in gastrocnemius weight happened in both tumour-bearing groups, being 22 % less in W and only 14 % in WM; comparing both tumour-bearing groups, in WM the muscle mass was 10 % higher than in W group. The muscle protein content reduced approximately 15 % in W, whereas WM group was similar to C. Tumour weight was not different between W and WM, although the relative tumour weight was around 23 % less in WM compared to W.

**Table 1:** Tumour growth effects on body composition, tissues weight rate and serum cachexia markers

	C (*n* = 12)	W (*n* = 12)	M (*n* = 12)	WM (*n* = 12)
Initial body weight (g)	65.8 ± 13.4	67.1 ± 11.3	67.2 ± 14.1	67.9 ± 11.2
Final body weight (g)	131.5 ± 14.3	119.5 ± 11.6*	135.2 ± 14.1	123.2 ± 11.5
Body water (%)	69.1 ± 1.8	73.6 ± 2.1***	69.1 ± 1.0	72.9 ± 2.7***
Body fat (%)	5.6 ± 1.9	2.9 ± 1.3***	6.3 ± 0.9	3.4 ± 1.6**
Lean body mass (%)	24.0 ± 2.9	21.3 ± 3.2*	23.5 ± 3.3	23.0 ± 3.4
Carcass protein (mg/g)	30.9 ± 1.8	26.4 ± 2.1***	30.9 ± 1.0	27.1 ± 2.7***
Collagen nitrogen (mg/100 g)	21.7 ± 5.6	14.2 ± 5.9**	19.2 ± 6.7	18.8 ± 4.8^#^
Non-collagen nitrogen (mg/100 g)	83.5 ± 7.2	88.4 ± 6.4	87.5 ± 6.2	84.8 ± 3.7
Total nitrogen (mg/100 g)	105.2 ± 7.1	100.1 ± 5.7	100.7 ± 11.0	103.3 ± 5.1
Muscle relative weight (%)	0.62 ± 0.05	0.48 ± 0.08***	0.62 ± 0.05	0.53 ± 0.07***
Muscular protein (μg/μL)	5.3 ± 0.2	4.5 ± 0.1*	5.5 ± 0.3	5.0 ± 0.2^#^
Tumour weight (g)	-	11.4 ± 3.1	-	10.1 ± 3.3
Tumour relative weight (g)	-	14.1 ± 4.0	-	10.9 ± 2.8^#^
Total plasma protein (g/dL)	5.4 ± 0.3	4.6 ± 0.4*	5.7 ± 0.9	5.0 ± 0.5^#^
Albumin (g/dL)	3.0 ± 0.1	2.3 ± 0.2***	2.9 ± 0.1	2.3 ± 0.3***
Globulin (g/dL)	3.2 ± 0.5	2.4 ± 0.3*	3.1 ± 0.9	2.6 ± 0.3

Legend: C, control rats; W, Walker 256 tumour-bearing rats; M, metformin-treated rats (33 mg/Kg); WM, Walker 256 tumour-bearing rats treated with metformin (33 mg/Kg). Number of animals per group = 12. Data are presented as mean ± SD. *p < 0.05 vs C; **p < 0.01 vs C; ***p < 0.001 vs C; #*p* < 0.05 vs W; ##*p* < 0.01 vs W

Total serum protein decreased by 15 % in W compared to C, whereas WM had similar content as C rats, and increased 11 % when compared to W. Albumin content reduced around 23 % in each W and WM group, while globulin concentration decreased only in W (22 % lower) (Table 1).

### Metformin decreases the tumour–induced protein degradation and ameliorates the protein turnover

**Fig. 1:**
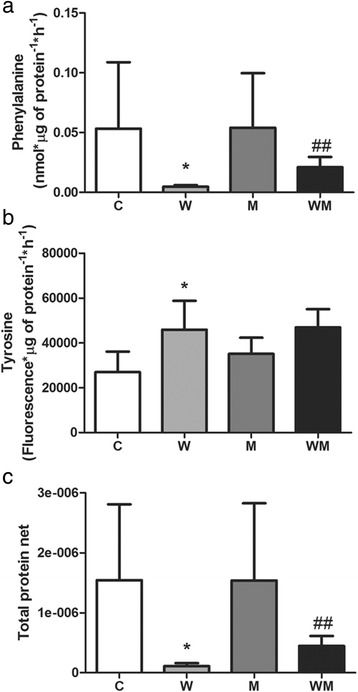
Metformin increases protein turnover in gastrocnemius muscle of Walker 256 tumour-bearing rats. **a** *Right* gastrocnemius muscle was pre-incubated for 30 min at 37 °C, in KHB buffer. Then, muscle was incubated for 2 h at 37 °C in KHB containing 0.5 μCi/mL L-[2,3,4,5,6-^3^H] Phenylalanine. Phenylalanine incorporated into muscle proteins represents protein synthesis rate. **b** *Left* gastrocnemius muscle was preincubated for 30 min at 37 °C in KHB buffer; then, additional incubation (2 h at 37 °C) in KHB containing 0.5 mM cycloheximyde; tyrosine released in incubation medium represents protein degradation rate. **c** Ratio between incorporated phenylalanine and released tyrosine was defined as protein turnover rate. Legend: C, control rats; W, Walker 256 tumour-bearing rats; M, metformin-treated rats (33 mg/Kg); WM, Walker 256 tumour-bearing rats treated with metformin (33 mg/Kg). Number of animals per group = 6. Columns represent mean ± SD. *p < 0.05 vs C; **p < 0.01 vs C; ##*p* < 0.01 vs W

Once many body parameters (Table 1) showed that tumour-bearing animals were cachectic, one might expect that protein synthesis was impaired associated to an increased protein degradation. To address this question, we analysed both protein synthesis and degradation. The tumour led to a decrease in gastrocnemius muscle protein synthesis, being 90 % less in W and 60 % in WM group when compared to C group (Fig. 1a). In parallel, tumour growth induced a significant increase in protein degradation in W group (90 % higher) and also in WM group (74 %; Fig. 1b). Despite having a tumour, the metformin treatment (WM group) induced a lower reduction in protein synthesis associated with a non-altered protein degradation (*p* = 0.0571). In this way, the ratio of muscle protein synthesis and degradation was deeply reduced in W (93 % less) whereas in WM this effect was modulated by metformin, being 4-times higher in WM than in W group (Fig. 1c).

### Proteasome is the major pathway for protein degradation in gastrocnemius muscle of tumour-bearing animals

**Fig. 2:**
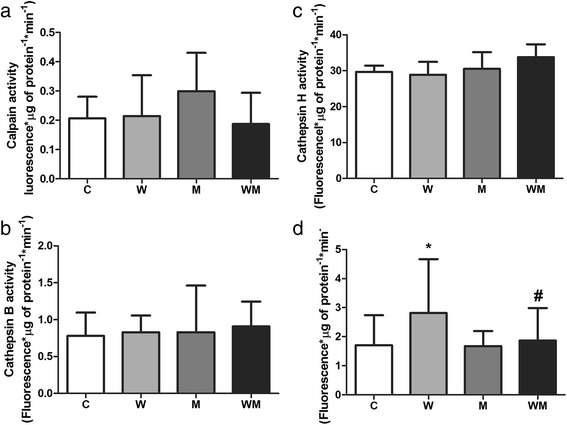
Muscle proteins are mainly degraded by proteasome pathway in Walker 256 tumour-bearing rats and metformin modulates this via. Enzyme activity of three different protein degradation pathways was analysed: (**a**) calcium-dependent pathway, (**b** and **c**) lysosomal pathway and (**d**) ubiquitin-proteasome pathway. Metformin modulates chymotrypsin-like activity in Walker 256 tumour-bearing rats bring the value close to C. Legend: C, control rats; W, Walker 256 tumour-bearing rats; M, metformin-treated rats (33 mg/Kg); WM, Walker 256 tumour-bearing rats treated with metformin (33 mg/Kg). Number of animals per group = 8. Columns represent mean ± SD. *p < 0.05 vs C; #*p* < 0.05 vs W

To address which protein degradation pathway was affecting the muscle mass and protein content in gastrocnemius, we analysed the three major protein degradation pathways. The calpain, cathepsin B and cathepsin H activities were similar in all experimental groups (Fig. 2a-c). Chymotrypsin-like activity was 64 % higher in W, while in WM this enzyme activity was similar to C (Fig. 2d). The comparison between tumour-bearing groups showed that metformin treatment modulated the chymotrypsin-like activity by 38 % less compared to non-treated group (Fig. 2d).

### Metformin minimises the effects of tumour growth in both synthesis and degradation pathways in gastrocnemius muscle

**Fig. 3:**
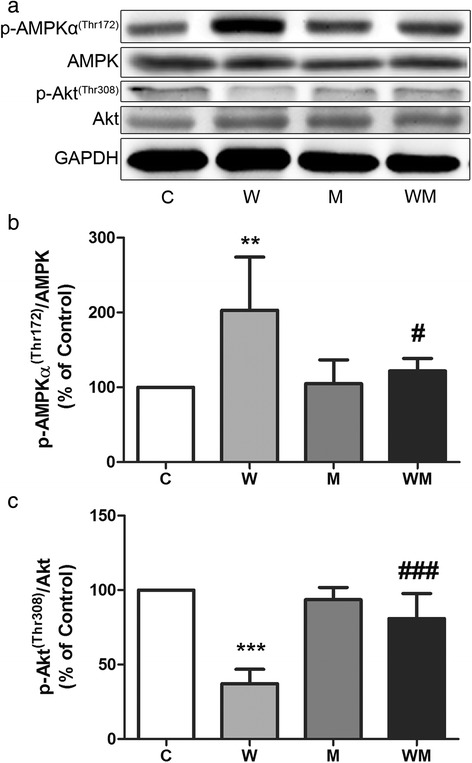
Metformin decreases inhibitory effects on protein synthesis in gastrocnemius muscle caused by tumour growth. Two different mTOR upstream proteins were analyzed by immunoblotting. **a** Representative image from at least four independent experiments. **b** AMPK was activated in W but not in WM, as showed by p-AMPK expression. **c** Akt phosphorylation was inhibited in untreated tumour-bearing animal (W) while in metformin-treated animals the activation was similar to control groups. Graphs (**b**) and (**c**) represent bands quantified and normalised by GAPDH expression. Values are relative percentage of C group, referred as 100 %. Legend: C, control rats; W, Walker 256 tumour-bearing rats; M, metformin-treated rats (33 mg/Kg); WM, Walker 256 tumour-bearing rats treated with metformin (33 mg/Kg). Columns represent mean ± SD. **p < 0.01 vs C; ***p < 0.001 vs C; #*p* < 0.05 vs W; ###*p* < 0.001 vs W

Metformin treatment greatly influenced the expression of proteins responsible for coordinating both synthesis and degradation processes in muscle. The ratio p-AMPK/AMPK increased two-fold in W while the WM showed similar value to C. The comparison between both tumour-bearing groups indicated that the effect of metformin treatment was remarkable; WM had 61 % lower p-AMPK/AMPK expression than W (Fig. 3a and b). The ratio of p-Akt/Akt was decreased approximately 63 % in W and was not affected in WM. On the other hand, the Akt activation in WM increased around 118 % compared to W (Fig. 3a and c).

**Fig. 4:**
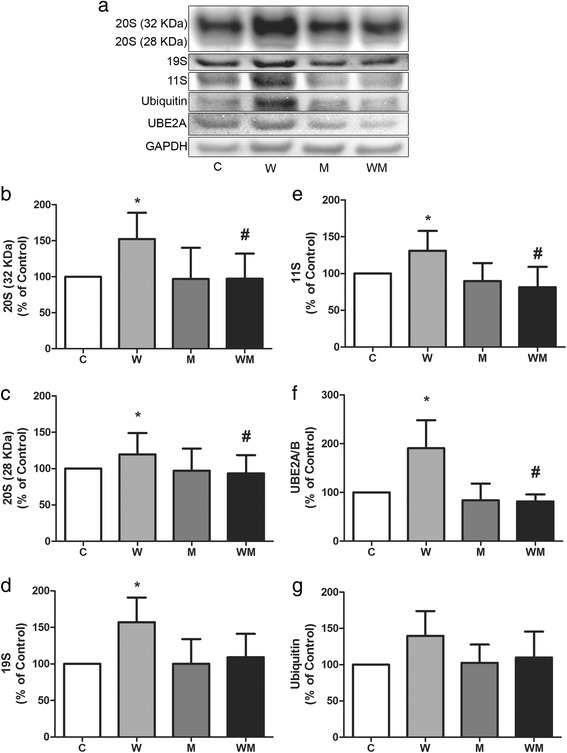
Expression of proteins of ubiquitin-proteasome pathway are increased in Walker 256 tumour-bearing rats and metformin is able to restore similar levels as C group. **a** Representative image from at least four independent experiments. Expression of proteasome 20S subunit, the 32 KDa subunit (**b**) and the 28 KDa subunit (**c**) were analyzed by immunoblotting. Regulatory proteasome subunits 19S (**d**) and 11S (**e**) likewise the ubiquitin-conjugating enzyme E2 (**f**) and ubiquitin itself (**g**) were also analysed by immunoblotting. Graphs from (**b**) to (**g**) represent bands quantified and normalised by GAPDH expression. Values are expressed as relative percentage of C group, referred as 100 %. Legend: C, control rats; W, Walker 256 tumour-bearing rats; M, metformin-treated rats (33 mg/Kg); WM, Walker 256 tumour-bearing rats treated with metformin (33 mg/Kg). Columns represent mean ± SD. *p < 0.05 vs C; #*p* < 0.05 vs W

Both proteasome 20S subunits (32 and 28 KDa) expression increased in W but not in WM (Fig. 4a, b and c). The 32 KDa subunit enhanced approximately by 52 % in W while in WM had no change compared to C. When compared to W, the expression of the 32 KDa subunit reduced around 36 % in WM (Fig. 4a and b). The 28 KDa subunit increased by 20 % in W and had no change in WM. Comparing both tumour-bearing groups, the expression of the 28 KDa subunit decreased approximately 22 % in WM (Fig. 4a and c). The 19S subunit expression increased around 57 % only in W group, while the WM had similar value compared to C (Fig. 4a and d). The 11S subunit expression increased approximately 31 % in W but had no change in WM (Fig. 4a and e). The UBE2A/B expression enhanced around 90 % in W compared to C whereas the WM group had no change (Fig. 4a and f). The ubiquitin expression was not affected either by the tumour or metformin treatment (Fig. 4a and g).

## Discussion

Cachexia is a common feature of many cancers, being responsible to worse prognosis in patients with different types of cancers [29], and is characterised as an involuntary weight loss, reduction of body fat and mainly the lean body mass [2]. We verified that metformin at a similar concentration to that prescribed to T2DM patients was able to minimise lean body mass loss and preserved protein content in gastrocnemius muscle. Many studies have shown that metformin can reduce cell proliferation in different tumour cell types [30, 31] and the results showed here are not exclusively due to a reduced tumour mass in metformin-treated animals.

Some tumours release factors that can change host metabolism and induce weight loss, mainly the lean body mass [32]. Besides lean body mass waste, tumour-bearing animals also showed a significant change in other parameters that indicates the establishment of the cachectic state, such as fat mass, water content and serum albumin concentration. Metformin treatment was able to ameliorate some of these effects possibly not decreasing any factor released by tumour but diminishing the impact of those factors on the host cells.

Tumour cells have an increased metabolic rate compared to non-tumour cells, probably because of the highly proliferative cell activity, which depends on a good provision of nutrients mobilised from host tissues. Due to that, metabolic tumour reprogramming has been described as a new cancer hallmark [33] and the possibility to explore these alterations using metformin treatment seems an excellent therapeutic opportunity. The ATP generation by aerobic glycolysis, which was described as the Warburg effect [34], is an efficient way by which tumour cells can get energy. On the other hand, these cells also need carbon and nitrogen source provided from other via such as tricarboxylic acid (TCA) cycle [35], whereas, parallel to the source of energy to tumour cells, the respiratory chain is still necessary to supply the biosynthetic pathways with precursor molecules such as lactate and alanine. The energy necessary to such neoplastic cells activities derives mainly from aerobic glycolysis, the increased rate of biosynthetic precursors and the maintenance of redox state are the most important needs of highly proliferative cells [36]. We have shown in our previous work that Walker 256 tumour-bearing animals have an increased lactate production while the metformin-treated tumour-bearing animals had a reduction of serum lactate concentration [20]. The lactate produced by tumour metabolism is carried to the liver and then converted back to glucose by gluconeogenesis, consuming three ATP molecules to generate glucose for each lactate molecule. In the same fashion, when muscle proteolysis occurs the generated alanine is transported by the bloodstream to the liver, where is converted to glucose. This reaction known as Cori Cycle could account for an additional loss of energy in cancer patients being at least 300 Kcal/day [37].

Animals bearing Walker 256 tumour have a reduced insulin secretion [38]. A decreased insulin-to-glucagon ratio indicates a starvation-like condition and, for this reason, the host cells or tissues are not able to prevent protein degradation, occurring lean body mass loss. We demonstrated that tumour growth led to a decrease in protein synthesis and increased protein degradation, reducing the gastrocnemius relative weight associated with a low muscle protein concentration as well as the lean body mass. The net protein loss in gastrocnemius muscle is likely due to a decreased Akt activation and an increased AMPK activation. Akt is phosphorylated and activated in a pathway triggered by insulin, which controls processes such as cell growth, metabolism, proliferation, glucose uptake, survival and angiogenesis [39]. Reduced circulating insulin means a reduction in Akt activation, leading to inhibition of mTORC1; whereas the phosphorylation by Akt, mTORC1 is activated, leading to protein synthesis process [40]. Conversely, AMPK is activated when the AMP:ATP ratio is high and inhibits mTOR, preventing additional energy expenditure. Tumour growth promotes lean mass spoliation in a manner to obtain substrates to keep growing and sustain the tumour cells high metabolic demands. In this sense, metformin treatment was efficient in minimising the protein depletion in gastrocnemius muscle.

We demonstrated that cytosolic degradation pathways, including calpain and cathepsin, were not activated in muscle of Walker 256 tumour-bearing animals, but the UPP is the main pathway by which muscle protein were degraded in these animals. The UPP handles muscle protein degradation in both human [41] and mouse [42] that have a tumour. In our previous work, it was demonstrated that Walker 256 tumour releases a cachectic factor similar to proteolysis-inducing factor (PIF) [43] and this factor was able to induce protein degradation and inhibit protein synthesis in myotubes cultured [43]. Although the PIF-like expression was not analysed in this work, probably the metformin led to a modulatory effect minimising the harmful effects over gastrocnemius tissue. PIF induces muscle cells to enhance UPP proteins expression and hence increasing protein degradation. A recent review pointed out the importance of ubiquitin ligases in the protein degradation process and how Akt activation stimulated by insulin was able to modulate it [7]. In our work, we have demonstrated that metformin treatment in tumour-bearing animals was able to keep UPP protein levels similar to the control group and, therefore, decreasing tumour effects in skeletal muscle. It is possible that metformin could maintain the insulin level close to the control group values; in our previous finds, we verified maintenance of insulin content in tumour-bearing rats treated with higher metformin dose. In this way, insulin activates Akt and, therefore, phosphorylate FoxO3a keeping it in the cytosol, inhibiting to act as a transcription factor for ubiquitin-ligases.

Overall, metformin treatment was able to decrease tumour growth (as the tumour/body weight ratio) and also able to decrease the harmful tumour effects on protein metabolism in skeletal muscle. As previously stated, metformin treatment is also known to inhibit mitochondrial complex I and, therefore, may be implicated in decreasing TCA intermediates [44]. In this scenario, as a probable mechanism of action, the metformin can be a toll on cancer treatment by decreasing the flux of molecules to biosynthetic pathways and the generation of reactive oxygen species (ROS) on tumour cells. However, ROS act as a double-edged sword in cancer progression; within certain limits, it can act as a growth-promoting agent, but in this case under metformin treatment, maybe there was a direct action to cell death [45]. It demonstrates the complexity of using the metabolic approach against tumour growth due to its high plasticity and heterogeneity [46].

Despite the fact that action mechanism of metformin is not completely understood, using it in laboratory animal models has shown remarkable antineoplastic effects [47]. However, further molecular data are needed to clarify this question, increasing the chance to use to use metformin as a therapeutic drug, because the evidences that metformin can be used as an adjuvant in cancer treatment are still incipient and its applicability needs to be confirmed in clinical trials.

## Conclusion

Taking all data together, we conclude that the cachectic state developed by tumour effects, mainly impairing the muscle tissue, as a consequence of higher activity of the ubiquitin-proteasome pathway, can be minimised under metformin treatment as this treatment ameliorated chemical body composition, reducing the ubiquitin-proteasome pathway, which decreased the protein waste. In summary, metformin remains a promising tool in cancer studies and how it could attenuate cachexia in tumour-bearing animals is a subject for further research.
